# Effects of *Astragalus Membranaceus* supplementation on oxidative stability of Cashmere goat

**DOI:** 10.1002/fsn3.1786

**Published:** 2020-09-01

**Authors:** Yulong Luo, Lin Su, Rina Su, Bohui Wang, Chang Liu, Zhenggang Wang, Lihua Zhao, Ye Jin

**Affiliations:** ^1^ College of Food Science and Engineering Inner Mongolia Agricultural University Hohhot China; ^2^ School of Agriculture Ningxia University Yinchuan China

**Keywords:** antioxidant ability, antioxidant enzymes, Cashmere goat, grazing style, oxidation

## Abstract

*Astragalus membranaceus* (AM) provides a rich source of polysaccharides that can act as powerful antioxidants, but their potential as feed ingredients in the lamb industry still rarely exploited. The objective of this study was to investigate the effect of dietary *astragalus membranaceus* supplementation on oxidative stability of goat muscles. Longissimus dorsi (LD) muscles from two groups of Cashmere goat (basal diet, C group; basal diet supplemented with 1% *astragalus membranaceus* root, AM group) were evaluated for lipid oxidation, myoglobin oxidation, activity of antioxidant enzymes, and antioxidant capacity. The results showed that color parameters in Cashmere goat of two feeding conditions were no significant difference (*p* > .05). In AM group, myoglobin (Mb) content was higher than C, while metmyoglobin (MMb) (*p* < .05) and malondialdehyde (MDA) (*p* < .01) were lower. Additionally *astragalus membranaceus* supplementation had a significant effect on superoxide dismutase (SOD) and catalase (CAT) (*p* < .001). In whole, the AM group goats presented a relatively higher antioxidant capacity than C. Especially, RSA and CUPRAC values of AM group goats had significantly higher than C (*p* < .05). Consequently, the AM group goats ingested abundant *astragalus membranaceus,* which enhanced the antioxidant capacity. Thus, it can eliminate free radicals and effectively inhibit oxidation.

## Graphical abstract

*Astragalus membranaceus* (AM) provides polysaccharides that can act as powerful antioxidants, and AM goats presented a relatively higher antioxidant capacity than the C (basal diet) goats

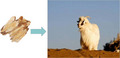

**Highlights**

- *Astragalus membranaceus* (AM) provides polysaccharides that can act as powerful antioxidants.
- AM group goats presented a relatively lower oxidation degree than the C goats.
- AM goats presented a relatively higher antioxidant capacity than the C goats.

## 1 INTRODUCTION

In livestock production, *astragalus membranaceus* (traditional Chinese herbal medicine) can improve its growth performance and antioxidant capacity, working as antioxidants to scavenge free radicals, and enhance the body's immunity and disease resistance. The advantages of Astragalus have no residue and side effects. In the previous studies, as a feed additive, the improvement of antioxidant capacity and meat quality of *astragalus membranaceus* are more common in pigs, chickens, geese, etc. But their application in goats has rarely been reported.

Inner Mongolia Cashmere goats are excellent variety, which can offer cashmere and meat. Now, the research on Cashmere goat is mainly focused on cashmere performance, rumen metabolism, and nutritional metabolism (Ansari‐Renani et al., 2011; Bai et al., 2017; Lei et al., 2017). However, the antioxidant properties of cashmere goats are rarely studied. Some researches found that Cashmere goat's cashmere and meat are not only affected by animal species, age, sex, feeding methods and other factors, but also closely related to its antioxidant capacity (Qwele et al., 2013; Zervas & Tsiplakou, 2011). Several researchers have shown that dietary supplementation can affect meat color, flavor, and oxidative stability (Rezar et al., 2017; Rossi et al., 2013; Schwarz et al., 2016). Poor feed structure can make organism excessive oxidation and speed up the aging of cashmere goats. It eventually leads to the production of cashmere and meat quality decline. Thence, antioxidants applied to livestock and poultry diets have become a cost‐effective regulation method for enhancing the antioxidant capacity and improving meat quality. Usually, polyphenol‐rich plants have been confirmed to improve the oxidative stability in different animal meat (Larraín, Krueger, Richards, & Reed, 2008; Nieto, Díaz, Bañón, & Garrido, 2010). Generally, improving the overall muscle's antioxidant status can decrease myoglobin oxidation extent and keep meat oxidative stability (Descalzo et al., 2007). Studying on the muscle antioxidant capacity of Cashmere goat can reflect its ability to resist stimuli, diseases, etc., which indirectly reflect the body's growth and development, the output of high quality fluff and meat quality.

Therefore, the aim of this study was to evaluate the effect of *astragalus membranaceus* supplementation on oxidative stability of Cashmere goats, and establish feeding recommendations by utilization of green feed additives to improve meat quality and extend shelf life in Cashmere goat production.

## 2 MATERIALS AND METHODS

### 2.1 Experiment site

This study was conducted in the livestock farm of Daqing area, which belongs to the south section of Yin Mountains, Inner Mongolia Province of China. The area is the typical temperate continental climate. The location lies within latitude 40°47′N and longitude 110°31′E at an attitude of 1,700 m above sea level. The mean annual rainfall of the area is approximately 380 mm.

### 2.2 Animals and management

Twenty‐four ewes (12 months old) with an average live weight of 27.86 ± 1.61 kg (mean ± *SD*) were equally assigned to the basal diet (control, C group) and basal diet supplemented with 1% *astragalus membranaceus* root (replace the corresponding proportion of corn stalk) (AM group) feed intake. According to Zhang et al. (2015), the optimal supplementation of *astragalus membranaceus* root in diet was 1%. The experimental period lasted 180 days from 5 April 2017 to 3 October 2017. The animals were kept for a stabilization period of 7 days before commencement of the experiment. All ewes were kept in individual pens (2.5 × 1 m^2^) with a feeder and waterer (access to water was ad libitum). All animals were fed twice daily at 8:00 and 18:00. 2 hr later, and the refusals of each animal were collected and weighed. Lambs were weighted every month before morning feeding. Ingredients and chemical composition of the basal diet are presented in Table 1. The supplement of *Astragalus membranaceus* root (diameter of less than 3 mm) was supplied by Shanxi Beiqi Industry Co., Ltd.

**TABLE 1:** Ingredients and chemical composition of the basal diet fed to Cashmere goat

Ingredients (%, as fed basis)	Basal diet
Corn stalk	27.76
Sunflower seed null	10.43
Cracked corn	39.62
Soybean meal	7.94
Linseed meal	11.99
Salt	0.6
Calcium hydrogen phosphate	0.56
Sodium hydrogen phosphate	0.20
Trace mineral mix	0.9
Total	100
Metabolizable energy (MJ/kg)	9.91
Chemical composition (%)	
Crude protein	11.85
Acid detergent fiber	19.73
Neutral detergent fiber	30.8
Calcium	0.56
Phosphorus	0.4

Note

Trace mineral mix: The diet of per kilogram provided Fe (FeSO_4_.7H_2_O) of 40 mg, Mn (MnSO_4_·H_2_O) of 20 mg, Zn (ZnSO_4_·H_2_O) of 40 mg, Cu (CuSO_4_·H_2_O) of 15 mg, Co (CoCl_2_) of 0.2 mg, and Se (NaSeO_3_) of 0.2 mg.

### 2.3 Animals and meat samples

The animal experiments were approved by the Committee of Animal Experimentation and were performed under the institutional guidelines for animal experiments of the College of Animal Science, Inner Mongolian Agricultural University, China. The C group (31.7 ± 1.61 kg) and AM group (33.1 ± 2.05 kg) were humanely harvested according to recommendations proposed by the European Commission (1997) to minimize the suffering of animals. After evisceration, carcasses were immediately split and cooled to 4°C, 24 hr postmortem (pH = 6.00 ± 0.30). Meat samples stored at 4°C were sliced from *Longissimus dorsi* (LD) for determination of meat color, Mb, and lipid oxidation. In addition, samples (10g) frozen at −80°C were retained for antioxidant enzyme activity assays.

### 2.4 Color measurement

The meat color was analyzed using a colorimeter (Minolta Chroma meter CR‐410, Konica Minolta Sensing Inc.) calibrated with a standard white plate (D65 light source; *Y* = 92.6, *x* = 0.3162, *y* = 0.3324) overwrapped with the applicable film, including lightness (L*), redness (a*), and yellowness (b*). Three colorimeter measurements were obtained on each sample. All measurement locations were taken on the skin side surface in an area free of obvious color defects.

### 2.5 Myoglobin concentration

The myoglobin concentration was determined according to method of Canto et al. (2016). Meat samples (5 g) were rinsed with physiological saline, and filter paper blotted up surface moisture. Plus 45 ml of ice cold sodium phosphate buffer (40 mmol/L, pH 6.8) and homogenated (4,000×*g*, 20s) after standing for 5 min. The absorbance of the filtrate was measured at 525 nm (A525) using a spectrophotometer (Persee TU‐1810, Persee Co. Ltd.). And sodium phosphate buffer was a blank. The myoglobin concentration was calculated using the following equation:

$$\text{Myoglobin}\;\left(\text{mg}/g\right)=\left[A_{525}/\left(7.6\;{\text{mM}}^{‐1}\;{\text{cm}}^{‐1}\times 1\;\text{cm}\right)\right]\times \left(17,000/1,000\right)\times 10$$

where 7.6 mM^−1^ cm^−1^ = millimolar absorptivity coefficient of myoglobin at 525 nm; 1 cm = light path length of cuvette; 17,000 Da = average molecular weight of myoglobin; and 10 = dilution factor.

### 2.6 Protein oxidation state

Protein oxidation state of meat sample was determined using a modified procedure of Kim et al. (2013). Samples (5 g) were blended with 5 volumes of cold phosphate buffer (0.04 M, pH 6.8) for 20 s in a homogenizer (Model XHF‐DF, Ningbo Scientz Biotechnology Co. Ltd.). The mixtures were centrifuged (3,000 *g*, 4°C, 30 min) after standing for 1 hr and the supernatant further clarified by filtration. The absorbance of filtrate was measured at 525, 545, 565, and 572 nm using a spectrophotometer (TU‐1810, Persee Co. Ltd.). The percent of oxymyoglobin and metmyoglobin was calculated using the formula (Krzywicki, 1982):

$$\text{OMb}\%=\left(0.882R_{1}‐1.276R_{2}+0.809R_{3}‐0.361\right)\times 100$$

$$\text{MMb}\%=\left(‐2.514R_{1}+0.777R_{2}+0.800R_{3}+1.098\right)\times 100$$

where *R*_1_ = ratio of the absorbance of filtrate at 572 nm and 525nm, *R*_2_ = ratio of the absorbance of filtrate at 565 and 525 nm, and *R*_3_ = ratio of the absorbance of filtrate at 545 and 525 nm.

### 2.7 Lipid oxidation

Lipid oxidation was assessed as thiobarbituric acid reactive substances (TBARS) and was measured by thiobarbituric acid reactive substances assay kit (A001‐1, Nanjing Jiancheng Bioengineering Institute, Nanjing, China) following the description of the manufacturer.

### 2.8 Activity of antioxidant enzymes

Sample (500 mg) was homogenized with ice cold saline (0.85%, 4.5 ml). Homogenate was centrifuged (4,000 *g*, 10 min, 4°C), and the supernatant was used to determine catalase (CAT), superoxide dismutase (SOD), and glutathione peroxidase (GPx) activity. Assay kits were used to determine CAT, SOD, and GPx activities.

CAT activity was measured according to the manufacturer's directions using a hydrogen peroxidase assay kit (A007‐1, Nanjing Jiancheng Bioengineering Institute). Absorbance at 550 nm was recorded spectrophotometrically (Persee TU‐1810, Persee Co. Ltd.), and one unit of CAT activity was defined as 1 mg tissue protein decomposed 1 μmol H_2_O_2_ per second at 37°C.

SOD activity was determined by measurement of hydroxylamine using a total superoxide dismutase assay kit according to the manufacturer's directions (A001‐1, Nanjing Jiancheng Bioengineering Institute). The absorbance at 550 nm was recorded spectrophotometrically (Persee TU‐1810, Persee Co. Ltd.). One unit of SOD activity was defined as the amount of enzyme in 1 ml of the reaction solution at 50% SOD inhibition at 37°C.

GPx activity was measured colorimetrically using a glutathione peroxidase assay kit according to the manufacturer's directions (A005, Nanjing Jiancheng Bioengineering Institute). Absorbance was recorded at 412 nm spectrophotometrically (Persee TU‐1810, Persee Co. Ltd.). One unit of GPx activity was defined as the amount of the enzyme capable of decomposing 1 μmol/L glutathione per minute at 37°C.

### 2.9 Antioxidant capacity

The antioxidant capacity was evaluated by three indicators, including total antioxidant capacity (T‐AOC), cupric reducing antioxidant capacity (CUPRAC), and free radical scavenging ability (RSA).

T‐AOC was measured by total antioxidant capacity assay kit (A015‐1, Nanjing Jiancheng Bioengineering Institute). Absorbance at 550 nm was recorded spectrophotometrically (Persee TU‐1810, Persee Co. Ltd.), and one unit of T‐AOC activity was defined as 1 mg tissue protein increased the absorbance of the reaction system by 0.01 per second at 37°C.

RSA of the samples was determined using procedure of Wen et al. (2015). Preparation of ABTS (2, 2′‐azinobis‐(3‐ethylbenzothiazoline 6‐sulfonate) reaction solution: 25 ml of 14 mmol/L ABTS mixed equal volumes of 4.9 mmol/L potassium persulfate solution and kept in the dark for 16 hr to produce ABTS cation radical (ABTS•+). The ABTS•+ solution was diluted with phosphate‐buffered saline (pH 7.4) an absorbance of 0.70 (±0.02) at 734 nm. The supernatant (as previously described) of the muscle extract was taken and (50 μl) mixed with 6 ml of ABTS•+, and equilibrated at 30°C for 6 min. The absorbance at 734 nm was read, with an equal volume of distilled water instead of the muscle extract as blank control. The percentage of inhibition of the ABTS•+ was calculated as follows.

$$\text{RSA}\%=\left[\left(A_{0}‐A_{1}\right)/A_{0}\right]\times 100$$

where *A*_0_ represents the absorbance of blank and *A*_1_ represents the absorbance of experimental group.

Cupric reducing antioxidant capacity (CUPRAC) was determined using procedure of Apak, Güçlü, Özyürek, and Karademir (2004) with modification. The supernatant of the muscle extract (50 μl) was added to the reaction system. The reaction system included 1 ml copper(II) chloride (10 mmol/L), 1 ml ammonium acetate(1 M), and 1 ml neocuproine in 96% ethanol (7.5 mmol/L). The reaction solution equilibrated at room temperature (22–25°C) for 1 hr. The absorbance was read at 450 nm, with an equal volume of distilled water instead of the muscle extract as blank. The CUPRAC was represented as ascorbic acid equivalents in mg/g muscle.

### 2.10 Statistical analysis

Twelve replications (*n* = 12) were utilized to data analyses in this study. All data were analyzed using the Statistical Package for the Social Science (SPSS Inc., version 19.0). Data of LD from goats were analyzed using one‐way analysis of variance (ANOVA) to examine the effect of *Astragalus membranaceus* on oxidative stability. The ANOVA tables obtained were further analyzed for the comparison of means by least significant difference (LSD) procedures. Mean values and relative standard deviation (RSD) were reported.

## 3 RESULTS AND DISCUSSION

### 3.1 meat color

The color of the Longissimus dorsi samples (LD) of Cashmere goat is presented in Table 2. *Astragalus membranaceus* supply increased L* in meat compared to C group (*p* < .05), but there was no effect of a* and b* value (*p* > .05). Polysaccharide is one of the main active ingredients of *astragalus membranaceus,* which can improve the redness of meat (Zheng et al., 2016). Zheng et al found the addition of *astragalus polysaccharide*s in diet can stabilize meat color, improving a* and reducing b* value. Sun also confirmed that *astragalus polysaccharide* can delay myoglobin oxidation and improve the meat color score (Sun et al., 2015). The meat color is directly related to the oxidized form and myoglobin concentration (Sánchez‐Escalante, Djenane, Torrescano, Beltrán, & Roncales, 2003). As the myoglobin was oxidized, the redness (a*) of the meat decreased (Suman, Hunt, Nair, & Rentfrow, 2014). Meanwhile, meat color stability is the wrestling between the main factors of the oxidation resistance and oxidation capacity, determining the meat color (Joseph et al., 2010).

**TABLE 2:** Meat color of Cashmere goat

Parameter	C group	AM group	*p*‐value
L*	35.72 ± 1.46	37.66 ± 1.98*	.029
a*	21.03 ± 1.37	21.58 ± 0.71	.320
b*	8.65 ± 1.41	7.45 ± 1.57	.114

Note

Values are expressed as mean and standard deviation (*n* = 12); basal diet, C group; and basal diet supplemented with 1% *astragalus membranaceus* root, AM group.

* *p* < .05.

### 3.2 myoglobin and lipid oxidation

The results of lipid oxidation and myoglobin (myoglobin (Mb), metmyoglobin (MMb), and oxymyoglobin (OMb)) oxidation are presented in Table 3. AM group demonstrated the lower malondialdehyde (MDA) than C (*p* < .001). It proved *astragalus membranaceus* supplementation can affect lipid oxidation in meat. Lipid oxidation can produce small molecules of secondary metabolites, such as aldehydes, ketones, and epoxy derivatives which can bind to myoglobin and promoted myoglobin oxidate to metmyoglobin, leading to surface discoloration (Lee, Phillips, Liebler, & Faustman, 2003; O’Grady, Monahan, & Brunton, 2001; Suman, Faustman, Stamer, & Liebler, 2007 and Faustman, Sun, Mancini, & Suman, 2010). Malondialdehyde (MDA), the main lipid peroxide, can usually reflect the degree of lipid peroxidation in the organization (Błaszczyk, Grucka‐Mamczar, Kasperczyk, & Birkner, 2008). In the present study, we found AM goats had lower degree of lipid peroxidation than C potentially due to a large number of antioxidant‐containing substances of *astragalus membranaceus*, such as polysaccharide, flavonoid, and polyphenol compounds. Additionally, the antioxidant substances were deposited in muscle tissue by long‐term feeding to AM goats, effectively inhibiting the tissue of the peroxide (Warren et al., 2008 and Kim et al., 2013).

**TABLE 3:** Lipid oxidation and myoglobin of Cashmere goat

Parameter	AM	C	*p*‐value
MDA (nmol/mgprot)	1.978 ± 0.834	4.811 ± 1.561**	.001
Mb (mg/g)	9.28 ± 1.51*	7.80 ± 0.90	.024
OMb (%)	37.66 ± 3.89	35.21 ± 4.65	.343
MMb (%)	29.84 ± 3.60	33.52 ± 2.29^*^	.02

Note

Values are expressed as mean and standard deviation (*n* = 12); basal diet, C group; basal diet supplemented with 1% *astragalus membranaceus* root, AM group.

Abbreviations: Mb, myoglobin; MDA, malondialdehyde; MMb, metmyoglobin;OMb, oxymyoglobin.

*p < .05; **p < .01.

The Mb and MMb were affected by *astragalus membranaceus* supplementation (*p* < .05), while the percentage of OMb was not affected. AM group demonstrated higher Mb than C group (*p* < .05). Some studies found that high Mb concentration in muscle was attributed to high proportion of type I fiber, resulting in greater a* value (Hwang, Kim, Jeong, Hur, & Joo, 2010; King et al., 2010; King, Shackelford, & Wheeler, 2011). In this study, the C group with low content of Mb and high MMb may have a great relationship with lipid oxidation (Faustman et al., 2010; Wongwichian, Klomklao, Panpipat, Benjakul, & Chaijan, 2015). The higher the degree of lipid oxidation, the more likely the myoglobin oxidized (Faustman et al., 2010; Luciano et al., 2009). C group has a higher degree of lipid oxidation than AM, which in turn causes OMb to be oxidized by free radicals generated during lipid oxidation (Attri, Jha, Choi, & Venkatesu, 2014; Estevez, 2011). MMb in AM group demonstrated lower value than C group (*p* < .05). Mancini reported that the red meat with more than 20% MMb relative content gradually darkened, reaching 50% changed into reddish brown, more than 70% became brown and lose sales value (Qwele et al., 2013). The consumer's favorite red meat color was mainly caused by the accumulation of oxymyoglobin (OMb), whereas the deterioration of meat color was related with the accumulation of metmyoglobin (MMb) (Zervas & Tsiplakou, 2011).

### 3.3 Antioxidant enzymes and capacity

The results of activity of antioxidant enzymes are presented in Table 4. The activities of the antioxidant enzymes (SOD and CAT) in AM group were significantly higher than C (*p* < .001), which were affected by *astragalus membranaceus* supplementation. This result aligns with work by Jin et al. (2017) who found supplemented ducks had a better antioxidant capacity than basic diet animals. Sun et al. (2015) also found that appropriate *Astragalus polysaccharides* in diet can significantly improve the antioxidant capacity of muscles and enhance immunity and disease resistance of chicken (Sun et al., 2015). In the present study, adding *astragalus membranaceus* in the diet can significantly increase SOD and CAT activity and reduce MDA content in muscle. Due to feeding appropriate amount of *astragalus membranaceus*, AM group increased the activity of antioxidant enzymes, which can clear free radicals and inhibit myoglobin oxidation (Fukai & Ushio‐Fukai, 2011; Sneddon et al., 2003).

**TABLE 4:** Antioxidant enzyme activity and antioxidant capacity of Cashmere goat

Parameter	AM	C	*p*‐value
SOD (U/mgprot)	100.46 ± 12.36***	69.02 ± 8.32	<.001
CAT (U/mgprot)	8.23 ± 1.68^***^	4.30 ± 0.72	<.001
GPx (U/mgprot)	3.85 ± 1.28	2.76 ± 0.91	.05
T‐AOC (U/mgprot)	1.25 ± 0.20	0.98 ± 0.44	.116
CUPRAC (mg/g)	2,457.93 ± 279.50*	2,156.56 ± 235.72	.016
RSA (%)	23.67 ± 2.68*	19.43 ± 1.67	.002

Note

Results are expressed as mean ± standard deviation; basal diet, C group; basal diet supplemented with 1% astragalus membranaceus root, AM group.

Abbreviations: CAT, catalase; CUPRAC, cupric reducing antioxidant capacity; GPx, glutathione peroxidase; RSA, ABTS•+ free radical scavenging ability; SOD, superoxide dismutase; T‐AOC, total antioxidant capacity.

*p < .05; ***p < .001.

The results of antioxidant capacity are presented in Table 4. AM demonstrated the higher CUPRAC and RSA activity than C (*p* < .01). Free radical can cause lipid oxidative chain reaction and make myoglobin in the Fe^2+^ oxidation of Fe^3+^, and RSA reflected all the antioxidant substances in muscle tissue free radical scavenging ability. This result confirmed that *astragalus membranaceus* supplemented in diet can enhance antioxidant ability. T‐AOC is a measure of muscle antioxidant capacity of the main indicators, reflecting the overall antioxidant capacity of the muscle instead of each antioxidant component contribution (Ghiselli, Serafini, Natella, & Scaccini, 2000). However, in the present study, T‐AOC was unaffected by the diet (*p* > .05).

On the whole, AM group goats have an advantage in suppressing oxidation, which may be related to its relatively rich antioxidant substances (Aouadi et al., 2014). These antioxidant substances can cleared oxygen free radicals and reduce MDA content in tissue to avoid cell damage caused by oxidative stress (Chen, Han, Yu, & Li, 2015; Luciano et al., 2011; Warren et al., 2008).

## 4 CONCLUSION

*Astragalus Membranaceus* supplementation influenced oxidative stability of Cashmere goat. Mb of AM group goats was higher than C (*p* < .05), while MMb (*p* < .05) and lipid oxidation degree lower (*p* < .01). As the antioxidant enzyme activities, CAT and SOD of AM were higher than C (*p* < .001). Among antioxidant capacity, the RSA and CUPRAC value of AM goats were significantly higher than C (*p* < .05). Consequently, the AM group goats ingest abundant *astragalus membranaceus*, which had an advantage in inhibiting oxidation. Thereby, the antioxidant capacity is higher than C group.

## CONFLICT OF INTEREST

The authors have declared that they have no conflicts of interest in this work.
